# From soil to plant: strengthening carrot defenses against *Meloidogyne incognita* with vermicompost and arbuscular mycorrhizal fungi biofertilizers

**DOI:** 10.3389/fmicb.2023.1206217

**Published:** 2023-06-14

**Authors:** Lukman Ahamad, Aashaq Hussain Bhat, Harendra Kumar, Aasha Rana, Md. Nurul Hasan, Ishtiaq Ahmed, Shakoor Ahmed, Ricardo A. R. Machado, Fuad Ameen

**Affiliations:** ^1^Section of Plant Pathology and Nematology, Department of Botany, Aligarh Muslim University, Aligarh, India; ^2^Department of Biosciences, University Center for Research and Development, Chandigarh University, Mohali, Punjab, India; ^3^Experimental Biology Research Group, Faculty of Science, Institute of Biology, University of Neuchâtel, Neuchâtel, Switzerland; ^4^Department of Zoology, J.S. University, Shikohabad, Uttar Pradesh, India; ^5^Department of Zoology, Faculty of Basic and Applied Sciences, Madhav University, Pindwara, Rajasthan, India; ^6^Zoological Survey of India, F.P.S. Building, Kolkata, India; ^7^Zoological Survey of India, New Alipore, Kolkata, India; ^8^Department of Botany and Microbiology, College of Science, King Saud University, Riyadh, Saudi Arabia

**Keywords:** arbuscular mycorrhizal fungi, sustainable agriculture, *Daucus carota*, disease management, *Meloidogyne incognita*, vermicompost

## Abstract

**Introduction:**

Sustainable agricultural practices for controlling crop pests are urgently needed to reduce the reliance on chemical pesticides, which have long-term detrimental effects on ecosystems. In this study, we assessed the effectiveness of arbuscular mycorrhizal fungi (AMF) and vermicompost (Vc) supplementation, alone and in combination, in mitigating the negative impacts of *Meloidogyne incognita* infestation on carrot (*Daucus carota* L.) growth, development, and physiology.

**Methods:**

We measured different plant growth parameters such as plant height and biomass accumulation, several plant physiological parameters such as the levels of photosynthetic pigments, phenolics, and the activity of defense enzymes such as peroxidases and polyphenol oxidases, and evaluated the severity of *Meloidogyne incognita* nematode infestation on plants treated or not treated with vermicompost (Vc) and/or arbuscular mycorrhizal fungi (AMF).

**Results:**

Our findings show that *M. incognita* significantly affects plant growth, biomass accumulation, and photosynthetic pigment and carotenoid content. The incorporation of Vc and AMF into the soil, either individually or in combination, significantly alleviates the negative effects of nematode infestation on carrot plants. This was accompanied by the induction of phenolic compounds and defense enzymes such as peroxidases (+15.65%) and polyphenol oxidases (29.78%), and by a reduction in the severity of nematode infestation on Vc and AMF-treated plants compared to nematode-infested plants. Principal component analysis (PCA) shows significant correlations between various of the studied parameters. In particular, we observed negative correlations between the application of AMF and Vc alone and in combination and disease severity, and positive correlations between plant growth, photosynthetic pigments phenol content, and activity of defense enzymes.

**Discussion:**

Our study highlights the relevance of cultural practices and beneficial microorganisms for the sustainable and environmentally friendly management of agricultural pests.

## 1. Introduction

Carrot (Apiaceae; *Daucus carota* L) is an economically important and nutrient-rich root vegetable crop cultivated in several countries worldwide. Its edible part, the taproot, is particularly rich in ß-carotene, vitamins, proteins and nutrients (Megueni et al., 2017; Fabiyi, 2021). One of the major threats to the production of carrots is root-knot nematodes (*Meloidogyne* spp., RKN). These minute worm-like animals create metabolic sinks in infested plants that limit photo-assimilates’ availability for plant growth. In addition, RKN colonizes the taproot, the root hairs and the lateral roots, forming a syncytium that disrupts the uptake of nutrients and water, thus causing substantial damage to carrot plants (Hussain et al., 2016; Khan et al., 2018). Moreover, by inducing galling and forking in the carrot roots, these nematodes render the infested carrot taproots unmarketable, thereby causing substantial direct economic losses (Prasad et al., 2014; Hussain et al., 2016). Therefore, finding sustainable and effective control measures against this agricultural pest is compulsory.

The fields infested with plant parasitic nematodes can be controlled by agricultural practices such as the use of nematicides, the application of biocontrol agents (Vagelas and Gowen, 2012), soil amendments (Asif et al., 2016, 2017), and other cultural practices such as crop rotation and the use of antagonistic plants (Hussain et al., 2011; Kayania et al., 2012). The application of chemical nematicides is highly effective in controlling root-knot nematodes, but their use is not recommended due to the huge detrimental effects on human health, the environment, and non-target organisms (Damalas and Eleftherohorinos, 2011). Currently, there is a worldwide swing towards eco-friendly, cost-effective and biocontrol management practices to combat nematode infestation for sustainable agriculture (Collange et al., 2011). One of the promising methods to combat nematodes is the application of vermicomposting and arbuscular mycorrhizal fungi (AMF).

Vermicomposting (Vc) is an eco-friendly biotechnological process that involves the combined action of earthworms and microbes to transform organic matter or waste to Vc nutrient-enriched products (Yadav et al., 2022). The microbes present in the gut of earthworms and feed-stock are accountable for the biochemical degradation of the organic matter (OM), while the earthworms are responsible for substrate breakdown and expanding the surface area accessible to microorganisms (Xu and Mou, 2016). Vermicompost nourish plants because it is rich in various nutrients, including calcium (Ca), phosphorus (P) and soluble potassium (K), which are essential for plant growth (Kumar et al., 2021). In addition, Vc is rich in humic acid compounds, nematicidal compounds (hydrogen sulfate, ammonia and nitrite) and hormones (cytokinins, indole acetic acid and gibberellins) that prevent nematode infestation (Oka, 2010; Khorshidi et al., 2013).

AMF are beneficial soil microorganisms that forms a symbiotic relationship with plants (Smith and Read, 2010). They have the potential to stimulate plant growth by improving the uptake of nutrients in return for photo-synthetic carbon (Vos et al., 2012). AMF decrease the severity of phytopathogenic nematodes (Fellbaum et al., 2012; da Silva Campos, 2020). Various mechanisms are involved in AMF-mediated biocontrol, including direct effects on the pathogen, such as competition for space and nutrients, or indirect effects, including the induction of plant defenses (Schouteden et al., 2015).

The current investigation explores the interactive effects of Vc and AMF applications on the growth, photosynthetic pigments, phenol contents and defense enzymes (peroxidase and polyphenol oxidase) in carrot plants when challenged with *M. incognita* nematodes. Our results reveal the great potential of Vc and AMF applications to ameliorate the negative impact of nematode infestation on carrot plants. Hence, our study highlights the relevance and feasibility of cultural practices and the use of beneficial microorganisms to control agricultural pests in eco-friendly and sustainable practices.

## 2. Materials and methods

### 2.1. Experimental setup

The experiment was carried out in a completely randomized block design (CRD). Plants were maintained at 28°C, 96% relative humidity, and 16/8 h photoperiod under greenhouse conditions. The light source in greenhouse was the sun during the daytime and an electric bulb during the early evenings. The following treatments were included: 1) Control, 2) *Meloidogyne incognita*, 3) AMF (*Funneliformis mosseae*) + *M. incognita*, 4) Vermicompost (Vc) + *M. incognita*, 5) Vc + AMF + *M. incognita*. Five independent replicates per treatment were analyzed.

### 2.2. Planting conditions

Plants were grown on loam soil collected in agricultural farms of the Aligarh district (Uttar Pradesh, India). Soil was crushed and passed through a 10-mesh sieve to obtain fine soil particles. The fine soil was mixed with farmyard manure in a ratio of 3:1, then autoclaved at 137.9 kPa pressure for 20 min. After sterilization, 1 kg soil mixture was filled in clay pots (15 × 8.5 × 13.5 cm). Prior to sowing, *Daucus carota* L. seeds cv. Red Rose were surface sterilized with 0.1% HgCl_2_ solution for 2 min followed by three times rinsing in autoclaved distilled water (DW). Seeds were purchased from the local market of Aligarh city, U.P., India. Five sterilized seeds were sown 2 cm deep in each pot. After emergence, the seedlings were thinned at the two-leaf stage to maintain one healthy plant in each pot. Plants were watered at regular intervals.

### 2.3. Collection of earthworms, leaf litter, and cow dung

The earthworms used in the experiments were purchased from a private vermiculture unit (Manglakanshi, Mahila Mandal F-1 Sai Vatika, Devpuri Raipur, Chhattisgarh), and were reared on partially decomposed cow dung under greenhouse conditions. A total of 10 worms were narcotized in 70% alcohol and preserved in 5% formaldehyde for species identification. The earthworm species were identified as *Eisenia fetida* Savigny based on taxonomic characters. The identification was carried out in the Zoological Survey of India (ZSI) (Kolkata, India). Fresh cow dung was collected from nearby cattle shed and stored for 1 week before its use for vermicomposting. The leaf litter (mixture of *Ricinus communis* L. and *Parthenium hysterophorus* L.) was collected from the vicinity of the Aligarh Muslim University, and chopped and dried prior to its use for vermicompost (Vc).

### 2.4. Preparation of vermicompost

Seven-day-old cow dung was mixed with chopped leaf litter in a 3:1 ratio respectively, and left in a pit (size 90 × 30 × 15 cm) for 3 weeks. During initial decomposition, organic material (OM) was mixed and turned upside down several times. In addition, water was added regularly to maintain the compost with moisture of 30%. After this time, a plastic container (45 × 30 × 30 cm) was filled up to 20 cm with the mixture of partially decomposed cow dung and leaf litter. This mixture was watered to reach 65–70% moisture and was kept for 24 h at 25–30°C to stabilize. One hundred mature *Eisenia fetida* worms were then released in 4 kg of the resulting compost (i.e., 25 worms/kg of compost). Then the top surface of the compost was covered with gunny bags and sprinkled with water to maintain 60–65% moisture for 60 days till the compost was ready to use. After 60 days, watering was stopped, the earthworms were sieved out, and the Vc was let to partially dry before its use for the experiments. 50 g of this Vc was used as a soil amendment for the experiments.

### 2.5. Preparation of sample for scanning electron microscopy and energy dispersive X-ray analysis

A small amount of Vc was fixed with a few drops of glutaraldehyde (2.5%) for 24 h at 4°C. After this, the sample was washed with 0.1 M phosphate buffer and centrifuged at 5,000–7,000 × g for 15 min at 4°C. The supernatant was discarded and the pellet was rewashed with 0.1 M phosphate buffer. This procedure was repeated thrice. The resulting pellet was fixed with a 1% OsO_4_ solution for 2 h at 4°C and washed thrice with 0.1 M phosphate buffer as described above. The pellet was then dried, mounted on SEM stubs and coated with gold. The mounts were examined for ultrastructural studies with a JEOL 6490LV low vacuum scanning electron microscope (Akishima, Tokyo, Japan). The Vc sample elemental composition was determined by Energy dispersive X-ray spectroscopy (EDX). After capturing the images of a particular sample, peaks of elements present in the sample were identified. After peak identification, the composition of elements present in the sample on a percentage basis was computed using an EDX spectroscope (INCA x-act, Oxford Instruments, Paris, France).

### 2.6. Sample preparation for Fourier transform infra-red spectroscopy

The vermicompost of cattle manure was processed for the FT-IR analysis. The raw Vc was dried at 40–50°C clean environments to avoid contamination for 4 to 5 h to remove the excessive moisture. The dried sample was crushed into a fine powder using a sterile mortar and pestle, and 1 mg of the sample was mixed with 100 mg of dry potassium bromide (KBr). The KBr-based pellets were compressed into thin disks using a hydraulic press (PCI Analytic, Mumbai, India) by establishing about 15-ton pressure. The disks were fixed into the sample holder of the FT-IR spectrometer (Thermo Nicolet 6700, Waltham, Massachusetts, United States), and the spectra were analyzed with a total of 32 scans against the KBr background. The FT-IR spectra were collected for the wave number 4,000–400 cm^−1^. Peak heights of spectra were measured using OMNIC software.

### 2.7. Collection and isolation of AM fungi from the rhizosphere of *Daucus carota* root

Soil samples collected from the carrot fields were processed using wet sieving and decanting techniques (Gerdemann and Nicholson, 1963). 100 g soil sample was dissolved into 1 L water, thoroughly shaken and left for 1 min to let the heavier particles settle down. The soil solution was first passed through a coarse sieve and then decanted on a series of sieves, i.e., 80, 150 and 300 meshes. The spores obtained on sieves were collected with water in separate beakers. The suspension of the spores was repeatedly washed with Ringer’s solution (NaCl 6 g/L, KCl 0.1 g/L and CaCl_2_ 0.1 g/L in distilled water of pH 7.4) to remove adherent soil particles from the spores and surface sterilized (Mosse and Hayman, 1971). The spore suspensions were poured on the filter papers placed in the funnels. The spores of similar shape and size were picked with the help of a camel hair brush under the stereomicroscope and put on a glass slide. Some of the collected AM fungal spores were mounted permanently on a glass slide in polyvinyl-lacto-glycerin (PVLG) containing polyvinyl alcohol 8.33 g, glycerin 5 mL and distilled water 50 mL (Koske and Tessier, 1983). The PVLG solution was mixed 1:1 (v/v) with Melzer’s reagent (Iodine 2.5 g; Potassium iodide 7.5 g; Chloral hydrate 100 g; Distilled water 100 mL) for the staining of fungal spores.

### 2.8. Morphological and molecular identification of arbuscular mycorrhizal fungi

The fungal species were identified morphologically under a 40X light microscope (Olympus SZ61). Spores (*n* = 3–5) of similar morphology were assembled on a strip in polyvinyl alcohol-lactic acid-glycerol (PVLG) mixture with Melzer’s reagent. The AMF spores were identified based on morphological characters such as spore color, dimension, the thickness of walls, number of walls and width of subtending hypha using the synoptic keys of Trappe (1982) and Schenck and Pervez (1990).

For molecular characterization, genomic DNA (gDNA) of the fungal spores collected above was extracted using PureLink^®^ Genomic Plant DNA Purification Kit (Thermo Fisher Scientific). Internal Transcribed Spacer (ITS) rRNA was amplified by PCR with the following components: 1 μL of gDNA, 0.5 μL of ITS1 (5′tccgtaggtgaacctgcgg-3′) and 0.5 μL of ITS4 (5′-tcctccgcttattgatatgc-3′) primers, 12.5 μL of Thermo Scientific DreamTaq Green PCR Master Mix and 10.5 μL nuclease-free dH2O (Bhat et al., 2023). The cycling conditions on the thermal cycler were set as follows: 1 cycle at 94°C for 5 min, followed by 37 cycles at 95°C for 60 s, 58°C for 45 s, 70°C for 60 s, and a final extension at 72°C for 10 min (Sebumpan et al., 2022). Sanger sequencing was performed in a bidirectional manner, and generated sequences were submitted to the National Centre for Biotechnology Information (NCBI) under the accession number OQ703041. The genomic sequences of fungi (*Funneliformis* sp.) closely related to the present sequence were searched using the Basic Local Alignment Search Tool (BLAST) of NCBI. The downloaded sequences were aligned with MUSCLE (v3.8.31) (Edgar, 2004), and evolutionary relationships were drawn by the Maximum Likelihood method based on the Tamura-Nei (T93 + G + 1) nucleotide substitution model in MEGA 11 (Tamura et al., 2021). The tree is drawn to scale, with branch lengths measured in the number of substitutions per site. Graphical representation and edition of the phylogenetic tree were performed with Interactive Tree of Life (v3.5.1) (Chevenet et al., 2006; Letunic and Bork, 2016).

### 2.9. Inoculum preparation of AM fungi

Spores of AM fungi were transferred to sterile paper cones. A solution of 2% chloramines-T and 0.025% streptomycin sulphate was poured drop by drop into each cone for 20 min (Hepper and Mosse, 1980). The spores were then washed thoroughly with sterilized distilled water. A pure culture of AM fungus (*Funneliformis* sp.) was maintained separately into each pot on *Chloris gayana* Kunth (Rhodes grass) grown in sandy loam soil mixed with river sand and farmyard manure in the ratio of 3:1:1 (v/v) respectively. The populations of AM fungus in the inoculums were assessed by the most probable number method (Porter, 1979). 50 g inoculum with soil was added around the seedling to inoculate 100 infective propagules of AM fungus per pot. The crude inoculum consists of soil, extra matrical spores and sporocarps, hyphal fragments and infective Rhodes grass segments. 50 g of AMF soil inoculum with approximately 200 spores was used for the experiments (Bhardwaj and Sharma, 2006). The crude inoculum of AMF consisted of soil, extra metrical spores and sporocarps, hyphal fragments and infected grass fragments.

### 2.10. Molecular and morphological identification of *Meloidogyne incognita*

Nuclear DNA was extracted from single specimens of nematodes as described by Bhat et al. (2021). The D2-D3 expansion segment of 28S rRNA was amplified by polymerase chain reaction (PCR) using the primers D2F: 5′-CCTTAGTAACGGCGAGTGAAA-3′ (forward) and D3R 5′-CAGCTATCCTGAGGAAAC-3′ (reverse) (Nadler et al., 2006). PCR reactions and cycling profiles were set as described by Rana et al. (2019). PCR products were purified, sanger sequenced and submitted to the NCBI under the accession number ON514606. The closely related sequences of other *Meloidogyne* species were downloaded from NCBI using the Basic Local Alignment Search Tool (BLAST) (Altschul et al., 1990). Resulting sequences were aligned with MUSCLE (v3.8.31) (Edgar, 2004), and phylogenetic relationships were constructed by the Maximum Likelihood method based on the Kimura 3–parameter (T92 + G) nucleotide substitution model in MEGA 11 (Tamura et al., 2021). The tree is drawn to scale, with branch lengths measured in the number of substitutions per site. Graphical representation and edition of the phylogenetic tree were performed with Interactive Tree of Life (v3.5.1) (Chevenet et al., 2006; Letunic and Bork, 2016). Perineal pattern arrangement was observed for the morphological characterization of nematodes in mature females.

### 2.11. Preparation of *Meloidogyne incognita* inoculum

Nematode inoculum was prepared by using the method described by Ahamad and Siddiqui (2021). Egg masses from severely infested roots of eggplants were picked for isolation of the infective juveniles (J2) using sterilized forceps. These egg masses were washed twice with dH2O and placed in sieves (9 cm diameter with 1 mm pore size) containing double-layered tissue paper and then placed in Petri dishes filled with distilled water deep enough to make a film with the egg masses. The whole setup was then incubated at 25 ± 1°C. The nematode J2s were collected in a culture flask daily, fresh distilled water was added as required in Petri dishes, and the process was repeated continuously to obtain the required number of nematode populations. The density of J2 nematodes in the suspension was assessed by direct counting under a light microscope. The volume of the suspension was adjusted to 100 nematodes per mL. 20 mL of this suspension were applied to each pot around the carrot seedlings (2000 freshly hatched J2s). Prior to inoculation, three holes were made gently around the roots so that J2 nematodes could reach the roots of seedlings and inoculum suspension was poured carefully into these holes.

### 2.12. Determination of plant growth characters

After inoculation, the plants were harvested after 90 days and cleaned with tap water to remove the adhered soil particles. Different growth traits such as plant length (cm), plant fresh weight (g), and shoot and root dry weight (g) were measured. The plant length was recorded by measuring the plant from the top of the shoot to the bottom of the root. To measure plant fresh weight, the roots were gently dried using blotting sheets before weighing them. Plant dry weight was evaluated in roots and shoots independently by cutting the plants at the root initiation zone with the help of a sharp knife to separate roots and shoots. Roots and shoots were kept in envelopes in an oven at 60°C for 7–10 days.

### 2.13. Determination of leaf photosynthetic pigment content

Chlorophyll and carotenoid content in fresh leaves were determined using the Mackinney method (MacKinney, 1941). Briefly, 1 g of freshly harvested leaves was mixed with 20 mL of 80% acetone and ground to a fine pulp using a mortar and pestle. The mixture was centrifuged at 5,000 × g for 5 min, and the supernatant was collected in 100 cm^3^ volumetric flasks. The residue was washed thrice with 80% acetone. Each washing was collected in the same volumetric flask, and volume was made up to mark, using 80% acetone. The absorbance was read at 645 and 663 nm for chlorophyll and 480 and 510 nm for carotenoid against the blank (80% acetone) on a spectrophotometer (Shimadzu UV-1700, Tokyo, Japan).

### 2.14. Determination of phenol content

The estimation of total phenol content was determined by the Folin-Ciocalteau reagent (Singleton and Rossi, 1965). Fresh leaves (2.0 g) were homogenized in 80% aqueous ethanol at room temperature and then placed in a cooled centrifuge at 10,000 × g for 15 min. Supernatants were collected and the remaining leaf pellets were re-extracted twice with 80% ethanol. All supernatants were pooled, put into evaporating dishes, evaporated to dryness at room temperature, and reconstituted in 5 mL distilled water. 100 μL of the resulting extract were mixed with 3 mL of distilled water, and 0.5 mL of Folin-Ciocalteau reagent. After 3 min, 2 mL of sodium carbonate (20%) was added. Samples were then thoroughly mixed and incubated for 1h. Then, absorbance at 650 nm was measured in a spectrophotometer (Shimadzu UV-1700, Tokyo, Japan). The results were expressed as mg catechol/g of fresh weight.

### 2.15. Determination of activities of peroxidase and polyphenol oxidase

To determine peroxidase (POX) and polyphenol oxidase (PPO) activities, 500 mg of fresh leaf tissues were homogenized in 5 mL of 50 mM phosphate buffer (pH 6.5) containing EDTA.Na2 (1 mM) and 1% polyvinylpyrrolidone. The homogenate was centrifuged at 15,000 × g for 10 min at 5°C, and the supernatant was used as an enzyme extract. Peroxidase (POX, EC 1.11.1.7) activities were measured by the method described by Chance and Maehly (1955) in fresh leaf samples. Pyrogallol phosphate buffer (3 mL), enzyme extract (0.1 mL) and 1% H_2_O_2_ (0.5 mL) were mixed in glass cuvettes, and changes in absorbance at 420 nm were measured at intervals of 20 s for 3 min using a spectrophotometer (Shimadzu UV-1700, Tokyo, Japan). The control group was prepared in a similar manner, but no enzyme extract was used. The peroxidase activity was expressed as U mg^−1^ fresh weight.

Polyphenol oxidase (PPO, EC 1.14.18.1) activities were measured using the protocol of Mayer et al. (1965) with minor modifications. The reaction mixture consisted of 200 μL of the enzyme extract and 1.5 mL of 0.1 M sodium phosphate buffer (pH 6.5). The reaction was started after adding 200 μL of 0.01 M catechol, and absorbance was recorded at intervals of 30 s for 3 min at 495 nm on a spectrophotometer (Shimadzu UV-1700, Tokyo, Japan). The control group was prepared in a similar manner, but no enzyme extract was used. The activity of polyphenol oxidase (PPO) was expressed as U mg^−1^ fresh weight.

### 2.16. Determination of root colonization by arbuscular mycorrhizal fungi

Arbuscular mycorrhizal fungi (AMF) root colonization was determined by the grid line intersecting method (Giovannetti and Mosse, 1980). AMF-inoculated roots were collected and washed gently with dH2O to remove soil particles. Roots were then cut into small pieces (1 cm), placed in KOH solution (10%) in a beaker, and autoclaved for 10 min at 103.4 kPa to remove the cytoplasmic content. The KOH solution was poured off and the root segments were rinsed in distilled water until no brown color appeared in the water. These root segments were then transferred in another beaker containing alkaline H_2_O_2_ at room temperature for 20 min for bleaching. Then, the roots were thoroughly rinsed with distilled water. The root segments were placed into a beaker containing HCl (1%) for 3 to 4 min. The solution was poured off and root segments were placed in another beaker with trypan blue lactophenol solution (0.05%). The root segments were observed under the stereomicroscope (Olympus, Mumbai, India) for mycorrhizal colonization. The per cent root colonization was calculated using the following formula:


$$\begin{array}{l} \mathrm{Percent}\;\left(\%\right)\;\mathrm{root colonization}=\\ \;\;\;\;\;\;\;\;\frac{\mathrm{Number of root segments colonized}\;\mathrm{by}\;\mathrm{AM}\;\mathrm{fungi}}{\mathrm{Total number of segments observed}}\times 100 \end{array}$$

### 2.17. Root galling and nematode density determination

The number of nematodes were determined by the Cobb’s sieving and decanting technique followed by Baermann funnel extraction (Southey, 1986). A total of 250 g of well-mixed soil from each treatment was processed. Nematode suspension was collected after 24 h, and the number of nematodes was counted in five aliquots of 1 mL of suspension from each sample. The mean of five counts was used to calculate the population of nematodes per kg of soil. To estimate the number of juveniles (J2s), eggs and females inside the roots, 1 g of roots was macerated in a Waring blender, and counts were made from the suspension thus obtained. The number of nematodes present in roots was calculated by multiplying the number of nematodes present in 1 g of the root by the total weight of the root. The number of galls and egg masses per root system was also counted.

### 2.18. Instruments

The chemical analysis of vermicompost was performed using a energy dispersive X-ray instrument from France and Thermo Nicolet 6,700, FT-IR spectrometer from United States. Biochemical studies were conducted using a Shimadzu UV-1700, UV-Vis spectrophotometer from Japan, which had a wavelength range of 190–1,100 nm and an accuracy of ±0.3 nm. The scanning electron microscope JEOL JSM-6490LV from Japan was utilized for ultrastructural studies.

### 2.19. Statistical analysis

At least five independent replicates were analysed for all the parameters measured. Data were analyzed using R software (2.14.0). Analysis of variance (ANOVA) was used to test the significance (*p* ≤ 0.05) of applied treatments. Least significant differences (LSD) and Duncan’s multiple range tests (DMRT) were employed to represent the significant differences between the treatments. Graphs were made by using SigmaPlot 14.0 software. Presentations of error bars in graphs showed the standard error (±SE). Principal component analysis (PCA) was done through OriginPro_2022 software.

## 3. Results

### 3.1. Occurrence and identification of AM fungi

**Figure 1:**
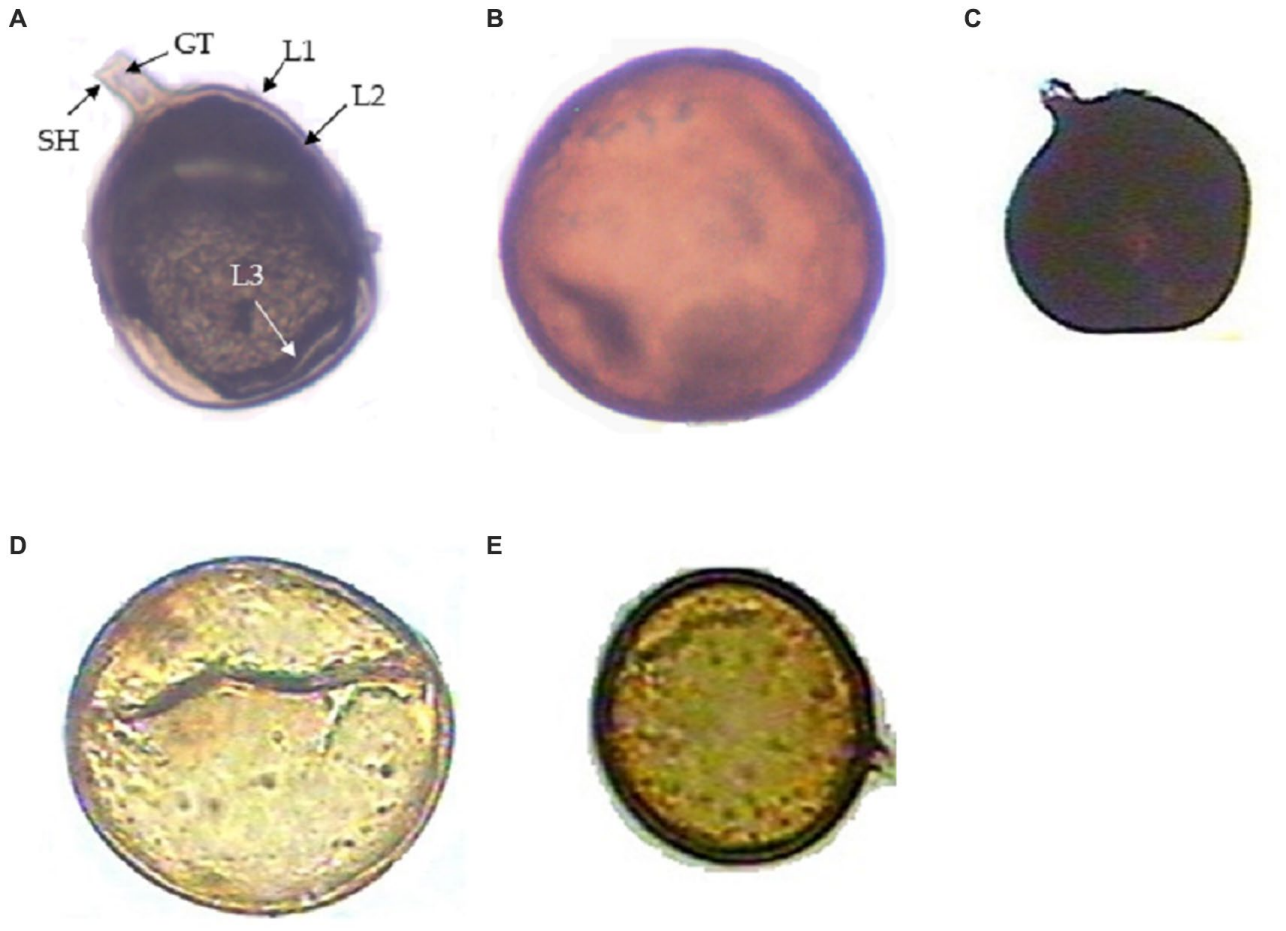
Different arbuscular mycorrhizal fungi (AMF) from the rhizosphere of *Daucus carota*. **(A)** *Funneliformis mossae* spore showing subtending hyphae (SH), germ tube (GH), wall structure-Layer 1, Layer 2, and Layer 3 designated as (L1), (L2), and (L3), respectively; **(B)** *Gigaspora* sp., **(C)** *Septoglomus* sp., **(D)** *Claroideoglomus* sp., and **(E)** *Acaulospora* sp.

From the soil samples, five genera of AMF were observed and identified morphologically: *Funneliformis* sp., *Gigaspora* sp., *Septoglomus* sp., *Claroideoglomus* sp. and *Acaulospora* sp. (Figures 1A–E). *Funneliformis* isolates possess visible septum, often cylindrical or funnel-shaped, and have subtending hypha. *Gigaspora* isolates bear irregular to rarely subglobose septum, spore wall consisting of two layers, stained dark red to red-brown in Melzer’s reagent. *Septoglomus* isolates display dark black colored spores visible when stained with Melzer’s reagent and having two- or three-layered walls, one subtending hypha. *Claroideoglomus* isolates show flexible, thin, colorless innermost layer; and bill-shaped, colorless subtending hyphae. *Acaulospora* sp. shows a wall structure with three layers, whitish-yellow in color, and possesses a granular germ layer with a beaded surface that reacts to Melzer’s reagent.

The number of spores of AM fungi also varied across treatments. *Funneliformis* sp. was the most abundant genus (4.3 spores/g of soil), followed by *Gigaspora* (3.01 spores/g of soil), *Claroideoglomus* sp. (2.6 spores/g of soil), *Acaulospora* sp. (2.38 spores/g of soil) and *Septoglomus* sp. (1.88 spores/g of soil) (Table 1). Of these AMF genera, *Funneliformis* sp. was used in the present investigation and was subjected to detailed morphological and molecular characterization.

**Table 1:** Occurrence of different arbuscular mycorrhizal fungi isolated from the rhizosphere of carrot roots.

S. No.	Arbuscular mycorrhizal fungi	Spore per 100 g soil
1	*Funneliformis mosseae*	430
2	*Gigaspora* sp.	301
3	*Septoglomus* sp.	188
4	*Claroideoglomus* sp.	260
5	*Acaulospora* sp.	238

**Figure 2:**
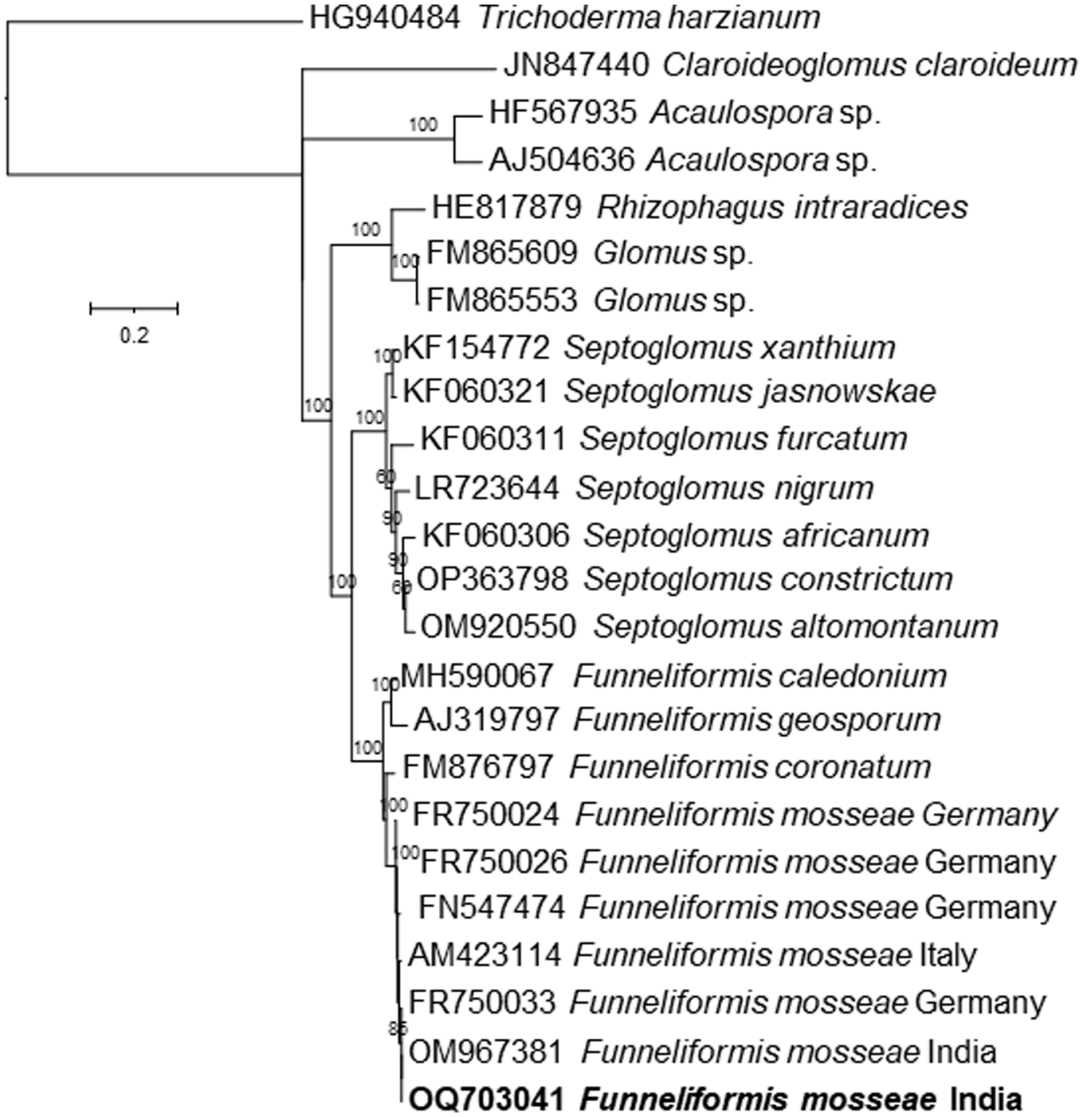
Phylogenetic tree based on ITS rRNA gene sequences of the *Funneliformis mosseae* isolated in this study and several related species inferred by using the Maximum Likelihood method based on the Timura-Nei Parameter Model. The percentage of trees in which the associated taxa clustered together is shown next to the branches. The tree is drawn to scale, with branch lengths measured in the number of substitutions per site.

Based on morphological characters, the *Funneliformis* sp. strain isolated in this study was identified as *Funneliformis mosseae*. It has visible septum, often cylindrical or funnel-shaped, and spore showing subtending hyphae, germ tube, and a three-layered wall (Figure 1A). The *Funneliformis* AM fungi were further characterized by analyzing the sequences of the ITS rRNA (OQ703041). The nucleotide sequences of the ITS rRNA of the fungal isolate used in this study show 100% similarity with the sequences of *F. mosseae* from different geographical regions of the world deposited in the NCBI. Upon alignment, no nucleotide difference was observed between the sequences analyzed. Phylogenetic reconstructions based on these sequences show that the AM fungi used in this study form a clade with previously described *F. mosseae* species from Germany, Italy, and India, and form a clade with *Funneliformis coronatum*, and together form a sister clade with *Funneliformis geosporum* and *Funneliformis caledonium* (Figure 2). Thus, morphological, molecular and phylogenetic studies confirm that the fungal isolate used in this study is *F. mosseae*.

### 3.2. Identification of root-knot nematodes

**Figure 3:**
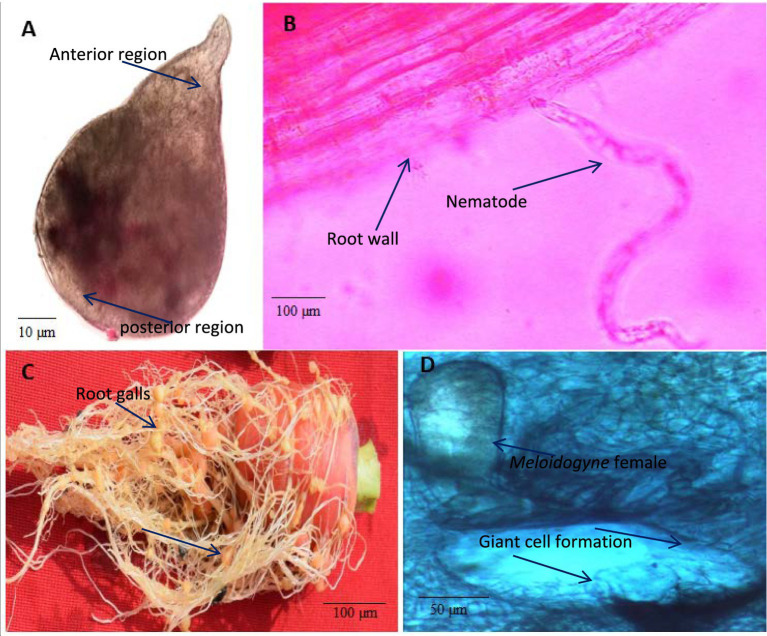
Light microscopy of *Meloidogyne incognita* nematodes on carrot plants. **(A)** *M. incognita* female, **(B)** A nematode juvenile (J2) invading root, **(C)** Root galling caused by *M. incognita* nematodes on carrot roots, **(D)** Transverse section (TS) of root gall showing the giant cell formation induced by the *M. incognita* female.

**Figure 4:**
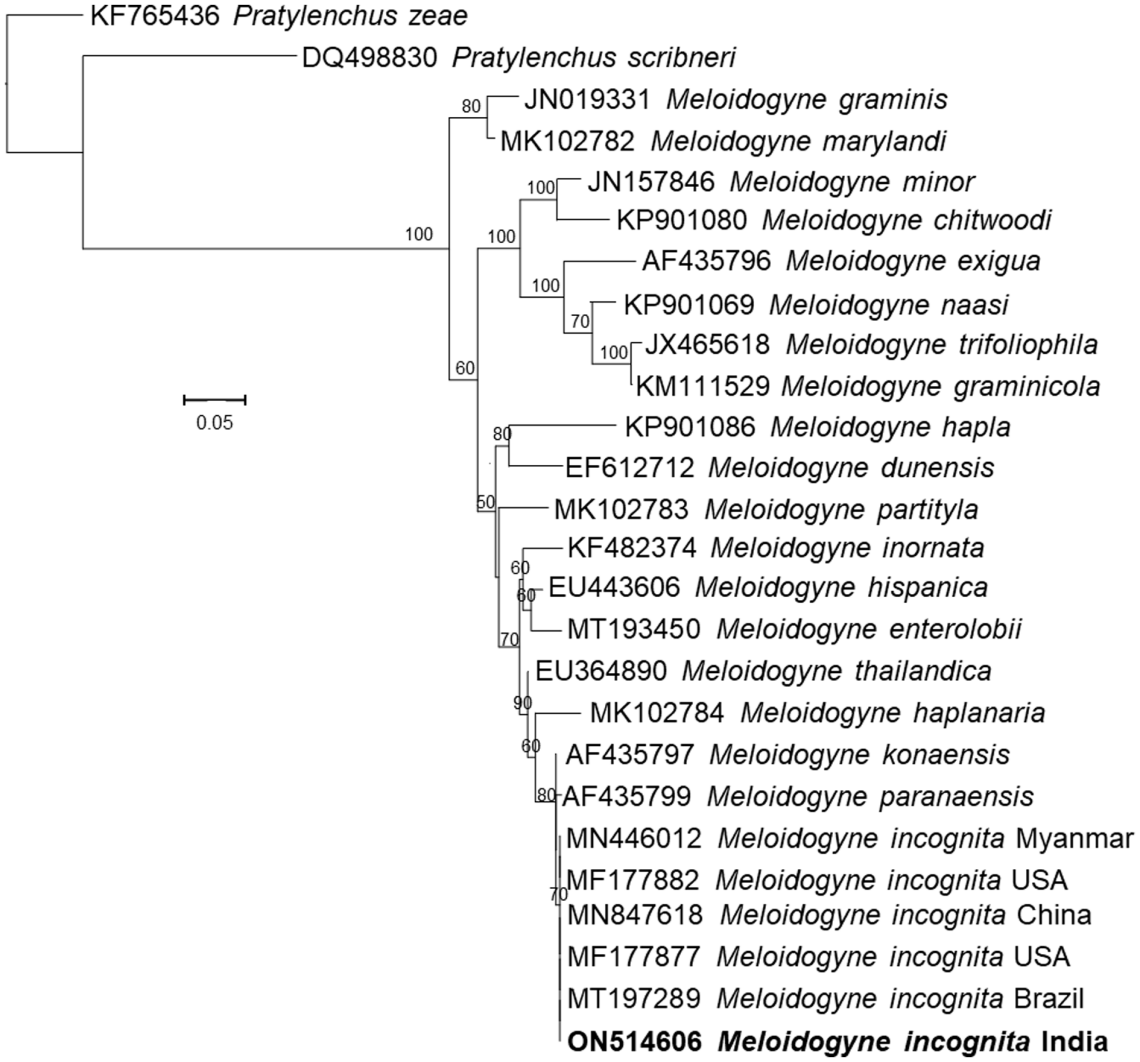
Phylogenetic tree based on D2D3 expansion segments of the 28S rRNA gene sequences of the *Meloidogyne incognita* isolated in this study and several related species inferred by using the Maximum Likelihood method based on the General Time Reversible model. The percentage of trees in which the associated taxa clustered together is shown next to the branches. The tree is drawn to scale, with branch lengths measured in the number of substitutions per site.

Morphologically, the perineal pattern of the adult females used in the present study showed an angular oval structure and an inverted V shape (Figure 3). This perineal pattern is similar to the previously described for *M. incognita* (Hunt and Handoo, 2009). These *Meloidogyne* nematodes were further characterized by analyzing the sequences of the D2-D3 region of the 28S rRNA gene (ON514606). The nucleotide sequences of the D2-D3 region of the nematodes used in this study show 100% similarity with the sequences of several other *M. incognita* nematodes from the United States, China, Brazil, Colombia, Myanmar, Vietnam and other regions of the world deposited in the NCBI. Upon alignment, no nucleotide difference was observed between the sequences analyzed. Phylogenetic reconstructions based on these sequences show that the nematodes used in this study form a clade with previously described *M. incognita* nematode species from China and USA, and together formed a group with other species (Figure 4). Thus, morphological, molecular and phylogenetic studies confirm that the nematode used in this study is *M. incognita*.

### 3.3. Energy-dispersive X-ray spectroscopy and scanning electron microscopy

**Figure 5:**
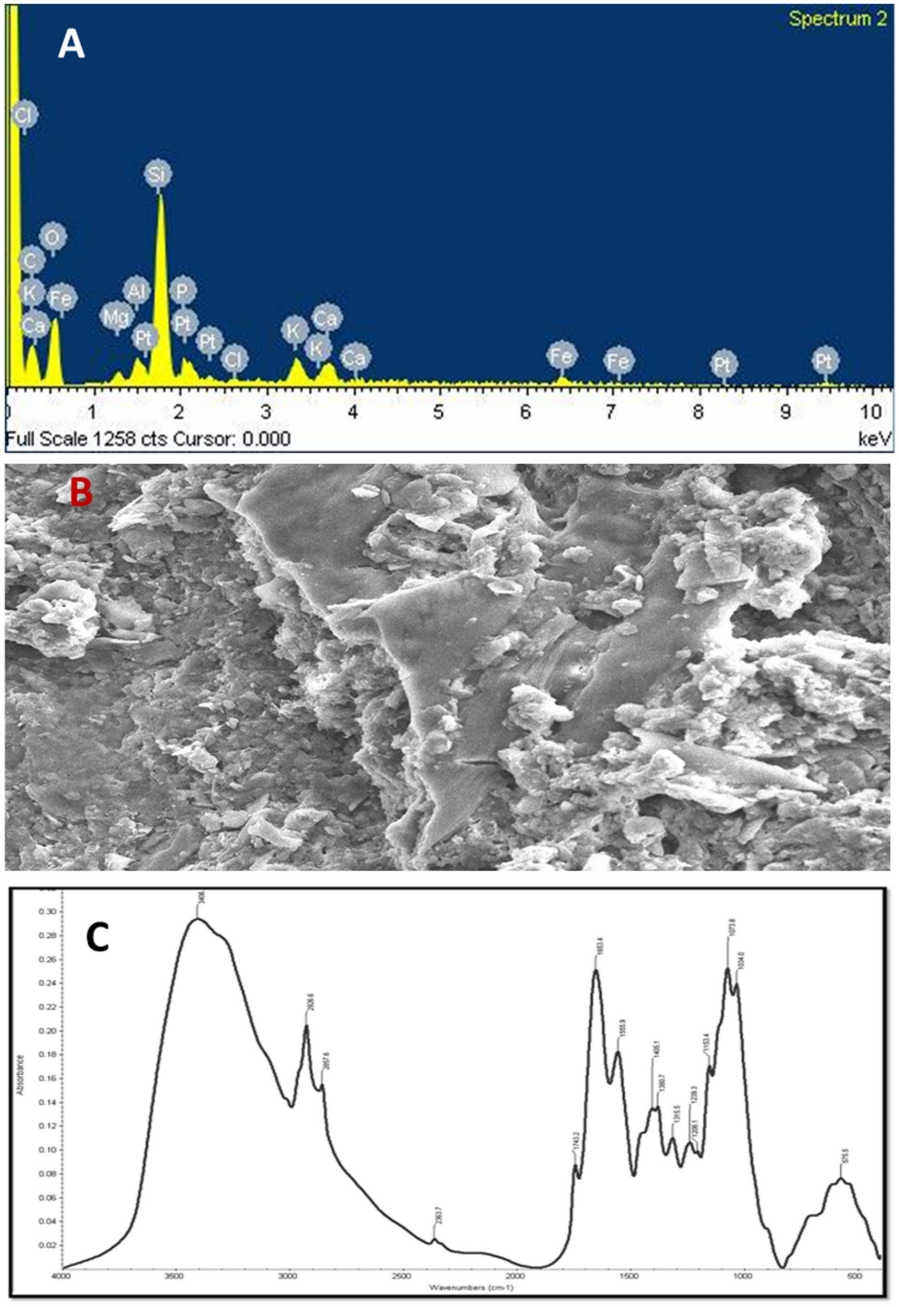
**(A)** Energy-dispersive X-ray (EDX) spectrum of vermicompost, **(B)** scanning electron microscope (SEM) analysis of vermicompost **(C)** absorbance spectrum and FT-IR dominant peaks of the vermicompost.

The observations of EDX analysis showed that O, N, Na, Mg, Al, Si, P, Cl, K, Ca, and Fe elements were found in the cow manure Vc. Moreover, oxygen (O) was the most predominant atom (46.29%) and sodium (Na) the least (0.25%) in the Vc (Figure 5A). Other elements, such as Fe, Mg, Ca, and K were also present in the of Vc at medium concentrations (Figure 5A).

SEM analysis was used to study the ultrastructural morphology of the prepared Vc. The SEM micrograph of the Vc sample revealed that the surface area of the Vc sample was larger and had more porosity, fragmentation, and granular structures. The highest proportion of surface area and the smaller particle size were recorded in the Vc sample. The micrographs showed a greater surface area with single particles packed together to form aggregates. Such aggregation is responsible for the uncertainty about the real surface area of Vc because the internal area was not fully accessible (Figure 5B).

### 3.4. Fourier transform infra-red spectroscopy

The FT–IR spectra were used to determine the compounds’ functional group(s) based on the peak values. The presence of primary amine was confirmed by the high absorption bands seen at 3,406 cm^−1^ as a result of the bonded N-H stretching (Figure 5C). The strong variable bands were observed at 2926.6 cm^−1^ to 2957.6 cm^−1^ due to asymmetric stretching of bonded C–H, which shows the presence of alkene (CH_2_) and alkane (CH_3_) groups. In addition, the bands observed at 2852.9 cm^−1^ to 2856.6 cm^−1^ due to the C–H symmetric stretch show the presence of alkene as a common biochemical constituent. The C–O bands were predominantly observed at 2363.7 cm^−1^ due to the carboxyl group, whereas the spectral band observed at 1743.2 cm^−1^ due to C=O stretch vibration corresponds to the presence of saturated aliphatic esters (Figure 5C).

The secondary structure of amine caused by N–H bending is observed at 1653.4 cm^−1^ to 1555.9 cm^−1^. The bands observed at 1405.1 cm^−1^ are caused by alcohols and phenols’ bonded C–O/O–H bending. The band observed at 1360.7 cm-^1^ due to C–H bending shows the presence of alkane. The IR spectrum seen at 1315.5 cm^−1^ as a result of S=O stretching shows the presence of sulphone. The bonded C–O absorbed IR spectrum at 1206.1 cm^−1^ shows the presence of ether functional groups. The strong bands observed at 1073.8 cm^−1^ to 1034.0 cm^−1^ due to C–O–C symmetric stretching shows the presence of polysaccharides. The presence of sulfide and disulfide compounds can be seen in the faint absorption bands at 575.5 cm^−1^, which may be caused by S-S stretching (Figure 5C).

### 3.5. Effects on plant growth parameters

**Figure 6:**
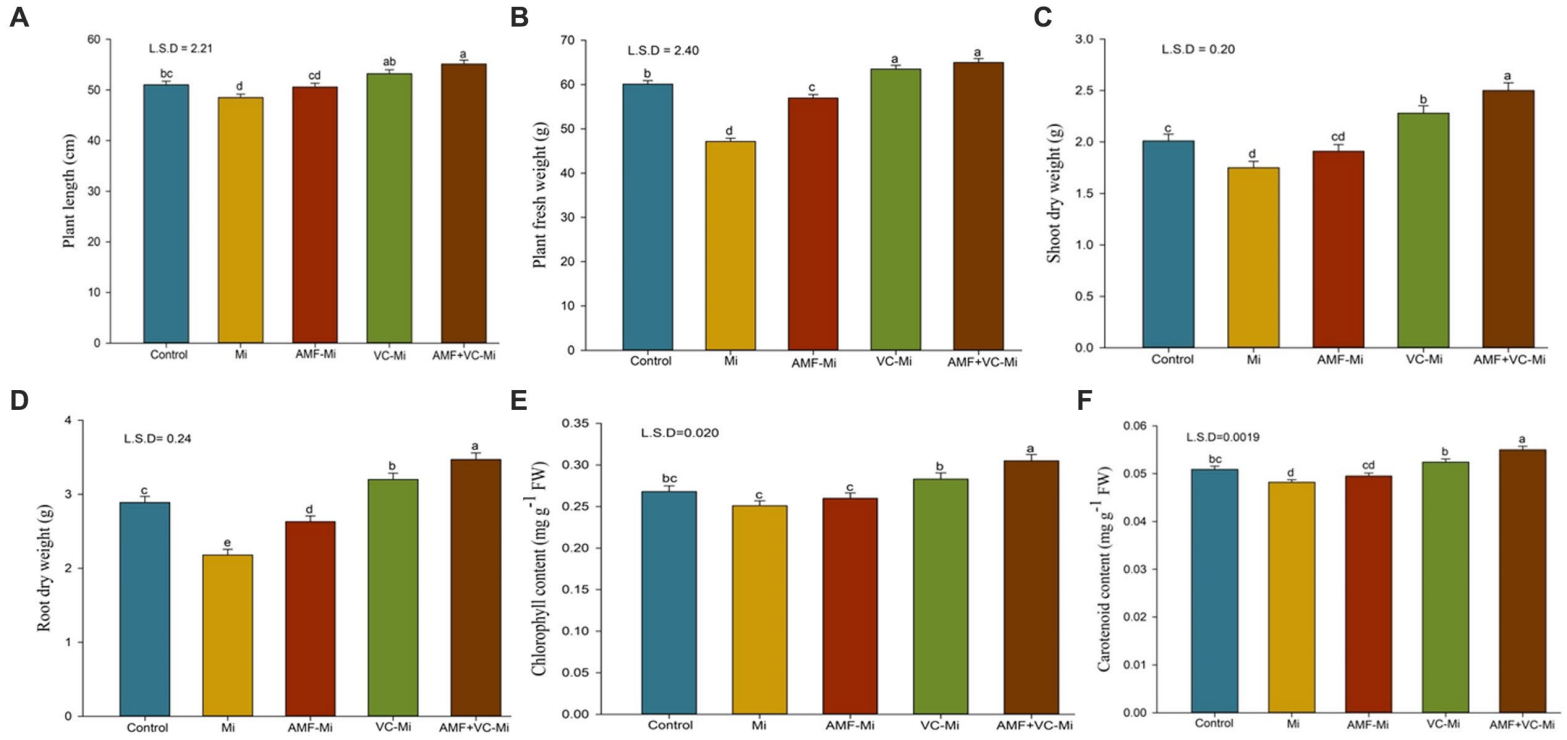
**(A–F)** Effects of arbuscular mycorrhizal fungi (*Funneliformis mosseae*) and vermicompost applications on different traits of plants infested or not by *M. incognita*. **(A)** Mean (±SEM) plant length. **(B)** Mean (±SEM) plant fresh weight. **(C)** Mean (±SEM) shoot dry weight. **(D)** Mean (±SEM) root dry weight. **(E)** Mean (±SEM) chlorophyll content. **(F)** Mean (±SEM) carotenoid content. AMF, Arbuscular-mycorrhizal fungi (*Funneliformis mosseae*); Vc, Vermicompost; Mi, *Meloidogyne incognita*; Error bars represent standard error (SE); Data present mean values of 5 replicates (*n* = 5).

*Meloidogyne incognita* significantly reduced plant growth parameters such as plant height, fresh weight, and shoot and root dry weight. The application of Vc and AMF (*F. mosseae*), both individually and in combination, significantly increased several plant growth parameters (Figures 6A–D). Interestingly, a more substantial positive effect was observed when Vc was applied to nematode-infested plants than when *F. mosseae* fungi were applied. Moreover, the combined applications of *F. mosseae* and Vc to nematode-infested plants increased the plant length by 13.60%, fresh plant weight by 37.85%, shoot dry weight by 42.85%, and root dry weight by 59.17% (Figures 6A–D).

### 3.6. Effects on chlorophyll and carotenoid content

A significant reduction in chlorophyll and carotenoid content was observed in *M. incognita*-infested plants (Figures 6E,F). A significant chlorophyll and carotenoid increase content was observed when *M. incognita*-infested plants were treated with Vc but not with AMF (*F. mosseae*). In addition, synergistic effects of Vc and *F. mosseae* treatments were observed. More specifically, the combined applications of both *F. mosseae* and Vc to *M. incognita*-infested plants increase chlorophyll and carotenoid content by 21.51 and 14.10%, respectively (Figures 6E,F).

### 3.7. Effects on phenol content, peroxidase and polyphenol oxidase enzyme activity

**Figure 7:**
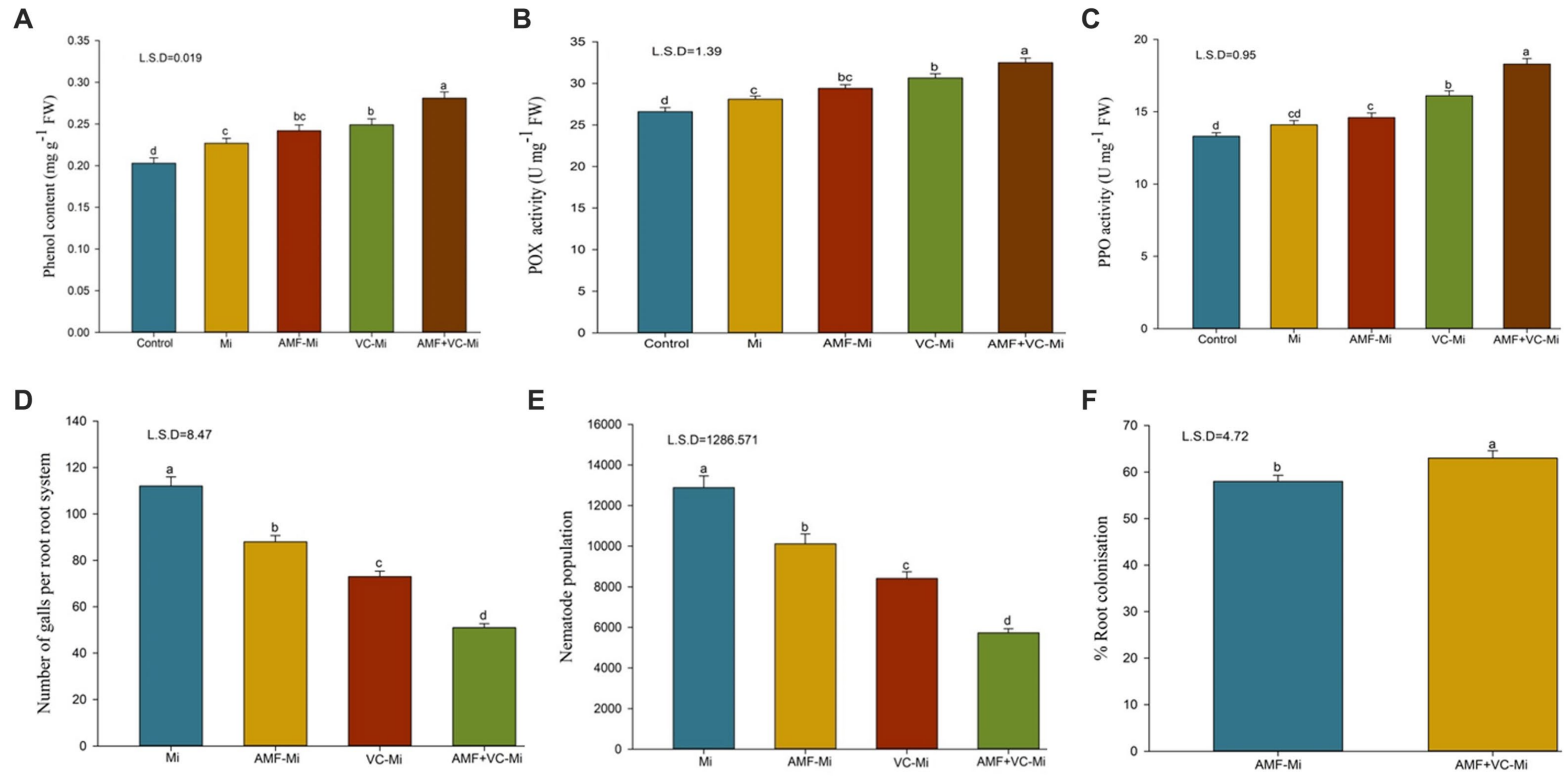
**(A–F)** Effects of arbuscular mycorrhizal fungi (*Funneliformis mosseae*) and vermicompost applications on different traits of plants infested or not by *M. incognita*. **(A)** Mean (±SEM) phenol content. **(B)** Mean (±SEM) peroxidase (POX) activity. **(C)** Mean (±SEM) polyphenol oxidase (PPO) activity. **(D)** Mean (±SEM) number of galls per plant. **(E)** Mean (±SEM) number of nematodes per plant. **(F)** Mean (±SEM) proportion of roots colonized by AMF (%). AMF, Arbuscular-mycorrhizal fungi (*Funneliformis mosseae*); Vc, Vermicompost; Mi, *Meloidogyne incognita*. Error bars represent standard error (SE); Data present mean values of 5 replicates (*n* = 5).

A significant induction of phenol content, peroxidase (POX) and polyphenol oxidase (PPO) enzyme activity was observed in *M. incognita*-infested plant (Figures 7A–C). When *M. incognita*-infested plants were treated with AMF (*F. mosseae*) or with Vc, phenol content, peroxidase (POX) and polyphenol oxidase (PPO) enzyme activity were further increased, although these effects were stronger in Vc-treated plants than in AMF-treated plants. In addition, synergistic effects of Vc and *F. mosseae* treatments were observed. More specifically, the combined applications of both *F. mosseae* and Vc to *M. incognita*-infested plants increase phenol content, POX and PPO activities by 23.78, 15.65, and 29.78%, respectively (Figures 7A–C).

### 3.8. Effects on root galling and nematode population with arbuscular mycorrhizal fungi colonization

**Figure 8:**
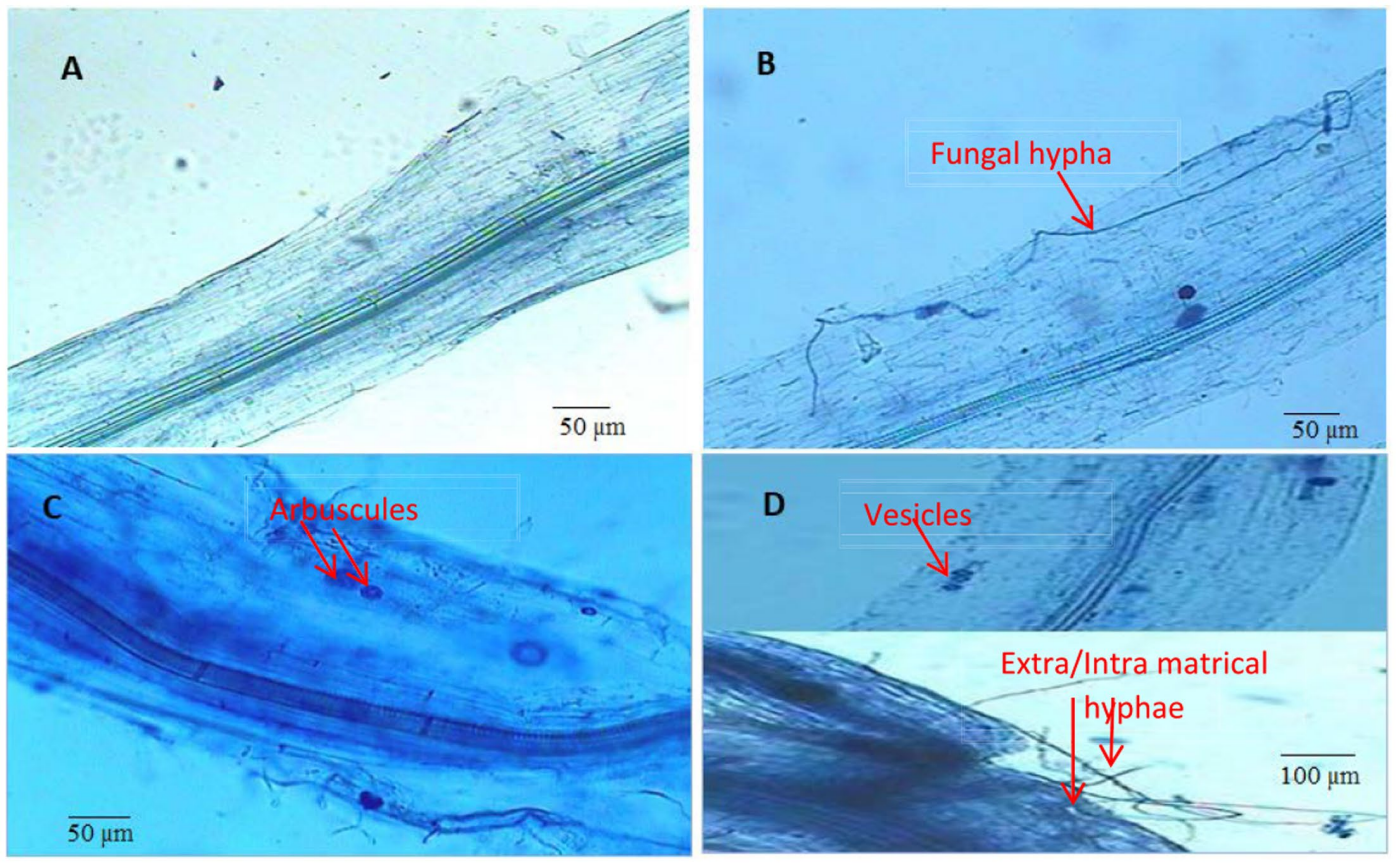
Root colonization by arbuscular mycorrhizal fungi (*Funneliformis mosseae*). **(A)** Control (without root colonization) **(B)** AMF treated plants (with root colonization) **(C,D)** AMF + Vermicompost treated plants (with root colonization).

A significant reduction in root galls and nematode population was observed when *M. incognita*-infested plants were treated with AMF or with Vc, alone or in combination. However, these effects were stronger in Vc-treated plants than in AMF-treated plants. In addition, synergistic effects of Vc and AMF treatments were observed (Figure 7). More specifically, the combined applications of both AMF (*F. mosseae*) and Vc to *M. incognita*-infested plants reduce root galling and nematode populations by 42.04 and 55.51%, respectively (Figures 7D,E). The mycorrhizal response of carrots inoculated with *M. incognita*, showed that the addition of Vc considerably (*p* = 0.05) boosted root colonization by *F. mosseae* (Figures 7F, 8). The application of Vc treatments resulted in a notable increase in mycorrhiza colonization in carrot plants that were infested with *M. incognita*. Among the treatments, the combination of AMF (*F. mosseae*) and Vc showed the highest percentage of root colonization (63%) in the presence of *M. incognita*, followed by the inoculation of *F. mosseae* alone (57%) (Figures 7F, 8).

### 3.9. Principal component analysis

**Figure 9:**
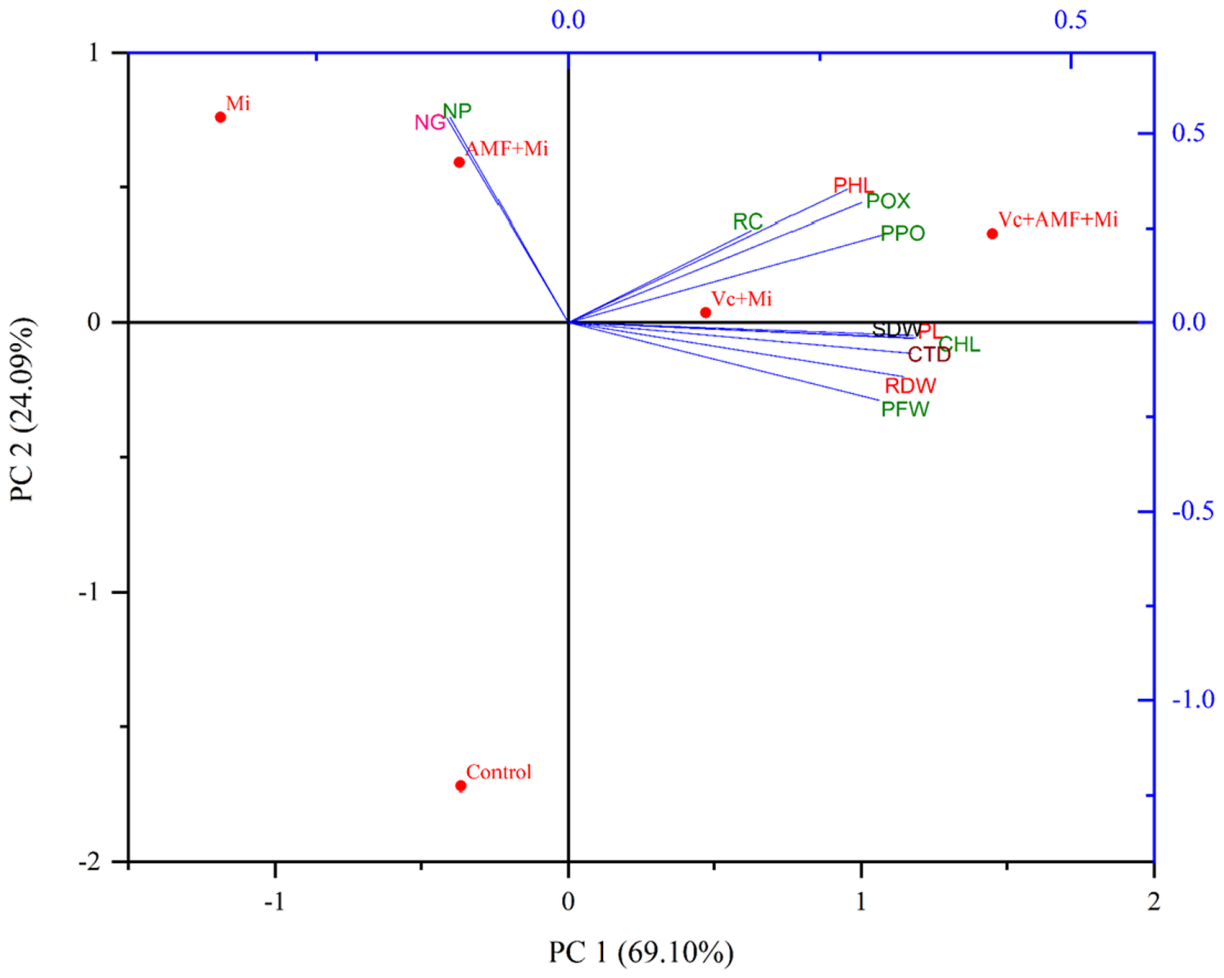
Biplots of principal component analysis (PCA) comparing the effects of arbuscular mycorrhizal fungi, vermicompost treatments and *M. incognita* infestation on different plant traits. PL, Plant length; PFW, Plant fresh weight; SDW, Shoot dry weight; RDW, Root dry weight; CHL, Chlorophyll content; CTD, Carotenoid content; PHL, Phenol content; POX, Peroxidase; PPO, Polyphenol oxidase; NG, Number of galls; NP, Nematode population; RC, Root colonization; Mi, *M. incognita*; AMF, Arbuscular mycorrhizal fungi (*Funneliformis mosseae*); Vc, Vermicompost.

Principal component analysis was carried out to study the effect of AMF (*F. mosseae*) and Vc on plant growth parameters (plant length, plant fresh weight, shoot and root dry weight), plant biochemical profiles (chlorophyll, carotenoid and phenol content), plant defense enzyme activities (POX and PPO), nematode disease severity parameters (root galling and nematode population). The PCA data showed an accumulated variability of 93.19%. The PC1 and PC2 displayed 69.10 and 24.09% (total 93.19%) of the novel information, respectively (Figure 9). AMF and Vc treatments, individually and in combination, show significant positive correlations with plant growth, photosynthetic pigment content, phenol contents and defense enzyme activities. Moreover, these latest plant traits showed a negative correlation with number of galls and nematode densities (Figure 9).

## 4. Discussion

In this study we show that the application of vermicompost and AMF to *M. incognita* attacked plants significantly alleviates the negative impact of these nematodes on plant growth. These effects are accompanied by the suppression of galling and nematode populations and a greater induction of phenolic compounds and defensive enzymes in vermicompost- and AMF-treated plants.

The application of Vc and AMF as soil amendments is a fast-emerging and encouraging area of research. Our results showed that the application of Vc and AMF to the soil significantly improved several plant growth parameters and greatly alleviated the negative consequences of *M. incognita* attack. A potential explanation for this observation is the induction of defense compounds that suppress nematodes and/or the increase of several macro- and micronutrients required for plant growth. In support of this hypothesis, previous studies also showed that the application of Vc in susceptible tomato plants increases root defense against root-knot nematodes by increasing the levels of defense compounds and by changing soil properties, which translates into better plant growth (Xiao et al., 2016). Furthermore, AMF significantly increased the growth through the induction of resistance against *M. incognita* in cherry tomatoes (Wang et al., 2022). In addition, AMF and Vc treatments improve plant defenses against other pathogens (Meng et al., 2021). In addition, some other studies show that Vc and AMF treatment enhances N, K, Ca, Zn, S and P contents in the soil, improving plant growth (Balliu et al., 2015). The AMF can significantly boost plant and soil characteristics in both root and shoot systems (Al-Hmoud and Al-Momany, 2017). Only a few reports are available on Vc and AMF, which showed beneficial roles in the growth and development of crops (Khorshidi et al., 2013; Khan et al., 2014; Oliveira et al., 2015; Shamshiri and Fattahi, 2016; Naeeni et al., 2017). The present study showed the AMF application alone and with the combination of Vc significantly increases the productivity of carrots and decreases the nematode infestation.

We also observed that AMF and Vc applications alone and in combinations increased the contents of plant pigments (chlorophyll and carotenoids). An increase in the pigments may be due to increase in nitrogen content of leaves of plants supplied with vermicompost (Yadav and Garg, 2015). Previous studies have shown that the Vc amendments in the soil increase the chlorophyll and carotenoid contents in plants, which in turn improves plant resistance against *M. incognita* (Xiao et al., 2016), and soil amendment with Rhizophagus irregularis (AMF) increases chlorophyll and carotenoid content of carrot plants infested with *M. incognita* (Ahamad and Siddiqui, 2021). Moreover, mycorrhizal inoculations enhance magnesium and phosphorus uptake in plants, which in turn increases chlorophyll content and overall performance of plants (Ait-El-Mokhtar et al., 2020). Vermicompost and AMF synergistically increase total chlorophyll content in plants when applied with *Rhizobium* (Rajasekaran and Nagarajan, 2004; Rajasekaran et al., 2006; Arumugam and Rajasekaran, 2010). Total chlorophyll content, fresh weight, and leaf area were higher in AMF-treated plants than in plants without AMF, but variations were notable under drought stress environments (Morte et al., 2000; Zhu et al., 2012; Sivakumar et al., 2020).

Our results also show that AMF and Vc applications alone and in combinations increased the phenolic contents and defense enzymes (POX and PPO). Several studies show that phenolics play an important role in plant resistance against biotic and abiotic stresses (Rehman et al., 2012; Tuominen, 2013). AMF effectively induces the accumulation of phenols and flavonoids, which are powerful antioxidants that act as free radical scavengers and reducing agents (Chen et al., 2013). The addition of AMF in the soil with or without *M. incognita* increases phenol contents in plants (Nagesh and Reddy, 2004). Soil amendments with Vc followed by inoculation of *M. incognita* increased the activities of defense enzymes (POX and PPO) in olive trees (Mohamed et al., 2019). Several studies report that the addition of compost increases plant defense against pathogens (Zhang et al., 1998). Similarly, AMF inoculations induces POX and PPO activities in cucumber plants infested with *M. incognita* (Choshali et al., 2019). AMF can improve plant tolerance to biotic and abiotic stressors and stimulate plant development under these conditions (Plassard and Dell, 2010; Alqarawi et al., 2014; Navarro et al., 2014). AMF immunization increases the nutritional value, physiology, quality and quantity of the plant products and pumps the biological activity, increasing the biochemical process (Yang et al., 2014; Canellas et al., 2015; Shi-Chua et al., 2019; Gao et al., 2020). In addition, the interaction between Vc and AMF may help speed up ^15^N acquisition through mineralization induced by vermicompost amendment and transfer from the soil to the plant (Liu et al., 2020).

With reference to root galling and nematode multiplication, when Vc was applied to carrot plants, a decrease in *M. incognita* population and the formation of galls was observed. Similar results were shown in tomato plants infected with *M. javanica* (Hemmati and Saeedizadeh, 2019). Moreover, nematode hatching is reduced when Vc exudates are applied (Mondal et al., 2021). Arugula Vc also decreases the reproduction factor of the nematode by 54.4 to 70.5% in infected tomato plants. It affects the expression of resistance genes and induces systemic resistance against root-knot nematodes (Rostami et al., 2022). Moreover, soil application of AMF fungi also reduced plants’ root galling and nematode population (Siddiqui and Akhtar, 2007). The mycorrhizal fungi could activate plant systemic resistance to resist possible nematode invasion (Nafady et al., 2022). Alternatively, mycorrhizal fungi may have a direct mechanism to suppress nematode root infection by competing for feeding and root space. The mechanisms by which this elicitation in the systemic defensive capacity of the roots occurs are related to the activation of genes that encode PR proteins, chitinases and enzymes that participate in reactive oxygen species (ROS) detoxification (whose accumulation occurs during hypertrophy and cell death by the nematodes) like glutathione S-transferase (GSTs)/superoxide dismutase (SOD), enzymes involved in the biosynthesis of lignin, and shikimate pathway which in turn, produces precursors of various aromatic secondary metabolites against nematodes (Schouteden et al., 2015; Sharma and Sharma, 2017; Balestrini et al., 2019). AMF colonization could reduce the density of *M. incognita* in soil (Wang et al., 2022). Soil amended with Vc increased the root colonization caused by AM fungi in the presence of *M. incognita* in carrot plants. Other studies also supported this statement that Vc application with AMF improves root colonization in tomato plants when infected with *M. incognita* compared to the individual application (Serfoji et al., 2010).

The surface area of the Vc was found to be larger, and it showed more porosity, fragmentation, and granular structures that are packed together to form aggregates as compared to pre-vermicompost. Complete internal area was not accessible due to these aggregates. The changes in surface morphology of pre- and post-vermicompost substrate mixtures and maturity were studied by SEM of Vc samples (Kumar et al., 2014; Unuofin and Mnkeni, 2014; Sharma and Garg, 2018; Srivastava et al., 2020). Irregular morphology in Vc samples with a high number of pores was observed by Kumar et al. (2014). Similar findings were observed in cation exchange capacity value in Vc samples (Pereira and Arruda, 2003; Ravindran et al., 2008). Disaggregation in the lignin matrix was followed by Hussain et al. (2016), it is because of the joint action of earthworms and microbes. Authors suggested that earthworms ingest and grind the substrate in their gizzard and pass it to the intestine, where enzymes and microbes carry out further degradation and disaggregation (Bhat et al., 2017). The EDX analysis in the present study showed the presence of various inorganic and organic elements in the cow manure vermicompost. The results of this study also corroborated with Zulhipri and Purwanto (2021), which showed that multiple elements of vermicompost were present in small and large proportions.

FT-IR analysis results indicate that this technique is suitable for the identification of the functional groups in Vc maturity and stability. Bhat et al. (2017) applied FT-IR spectra to analyze the mineralization of organic matter and degradation of complex aromatics (lignin, polyphenols) into simpler compounds (carbohydrates, lipids) by earthworms. The band height reduction was observed at 3,100–3,600 cm^−1^ in the Vc samples compared to the raw waste. Moreover, Sen and Chandra (2007) applied an estimate of humic acid derived from sugar industry wastes, which showed the broadband at 3,400–3,300 cm^−1^. Ravindran et al. (2008) identify the chemical structural changes in the control and final Vc mixtures of solid waste (animal fleshing) produced from the leather industry by applying FT-IR spectroscopy technique. FT-IR spectra observed a reduction of aliphatic compounds in the final Vc mixture. Deka et al. (2011) confirmed that FT-IR spectra of final Vc mixtures increased nitrogen compounds and decreased aliphatic and aromatic compounds compared to the pre-Vc waste mixtures. This indicates the degradation of bio-waste during the vermicomposting process. Lv et al. (2013) applied FT-IR spectra on water extractable organic matter (WEOM) extracted from the vermicomposting process of cattle dung, and observed a decreasing trend of aliphatic C–H stretching at 2936–2958 cm − 1 that confirms lipids and carbohydrate degradation. Similar findings were observed in FT-IR spectroscopy analysis of Parthenium mediated Vc (Rajiv et al., 2013). Ravindran et al. (2013) observed the appearance of COO groups and relative reduction in OH, CH_3_ and CH_2_ groups in Vc prepared from biodegradation of fermented animal fleshing mixed with leaf litter and cow dung using earthworm, *Eudrilus eugeniae*.

## 5. Conclusion

The root-knot nematode, *Meloidogyne incognita* causes significant damage to carrots under greenhouse and field conditions. Supplementation of Vc and AMF alone and in combination, enhanced plant growth, chlorophyll, carotenoid, and phenol contents, and increased in the defense enzyme activities. In addition, mycorrhizal root colonization was also increased when plants were amended with Vc in the presence of *M. incognita*. Vc and AMF soil amendments in carrot plants suppressed root galls and nematode populations. The results of the present study, therefore, strongly support the use of Vc and AMF against *M. incognita*. Hence, Vc along with AMF could be an efficient alternative to promote sustainable plant production under biotic stress conditions.

## Data availability statement

The datasets presented in this study can be found in online repositories. The names of the repository/repositories and accession number(s) can be found in the article/supplementary material.

## Author contributions

LA, AB, and AR: conceptualization, formal analysis, investigation, and writing—original draft. LA, AB, AR, and HK: data curation and methodology. RM and FA: funding acquisition. LA, AB, AR, RM, and FA: project administration. HK, MH, SA, RM, and FA: resources. LA, AB, AR, and RM: supervision and validation. LA, AB, AR, HK, MH, and SA: visualization. LA, AB, AR, HK, MH, SA, RM, and FA: writing—review and editing. All authors contributed to the article and approved the submitted version.

## Funding

The work of RM is supported by the Swiss National Science Foundation (Grant 186094 to RM). The work of AB is supported by Swiss Government Excellence Scholarship (Grant Nr. 2021.0463 to AB). The authors also extend their appreciation to the Deputyship for Research and Innovation, Ministry of Education in Saudi Arabia for funding this research work through the project no. (IFKSUOR3-005-2).

## Conflict of interest

The authors declare that the research was conducted in the absence of any commercial or financial relationships that could be construed as a potential conflict of interest.

## Publisher’s note

All claims expressed in this article are solely those of the authors and do not necessarily represent those of their affiliated organizations, or those of the publisher, the editors and the reviewers. Any product that may be evaluated in this article, or claim that may be made by its manufacturer, is not guaranteed or endorsed by the publisher.
