# Corrigendum: From soil to plant: strengthening carrot defenses against *Meloidogyne incognita* with vermicompost and arbuscular mycorrhizal fungi biofertilizers

**DOI:** 10.3389/fmicb.2023.1279879

**Published:** 2023-09-04

**Authors:** Lukman Ahamad, Aashaq Hussain Bhat, Harendra Kumar, Aasha Rana, Md. Nurul Hasan, Ishtiaq Ahmed, Shakoor Ahmed, Ricardo A. R. Machado, Fuad Ameen

**Affiliations:** ^1^Section of Plant Pathology and Nematology, Department of Botany, Aligarh Muslim University, Aligarh, India; ^2^Department of Biosciences, University Center for Research and Development, Chandigarh University, Mohali, Punjab, India; ^3^Experimental Biology Research Group, Faculty of Science, Institute of Biology, University of Neuchâtel, Neuchâtel, Switzerland; ^4^Department of Zoology, J.S. University, Shikohabad, Uttar Pradesh, India; ^5^Department of Zoology, Faculty of Basic and Applied Sciences, Madhav University, Pindwara, Rajasthan, India; ^6^Zoological Survey of India, F.P.S. Building, Kolkata, India; ^7^Zoological Survey of India, New Alipore, Kolkata, India; ^8^Department of Botany and Microbiology, College of Science, King Saud University, Riyadh, Saudi Arabia

**Keywords:** arbuscular mycorrhizal fungi, sustainable agriculture, *Daucus carota*, disease management, *Meloidogyne incognita*, vermicompost

In the published article, there was an error in affiliation 2. Instead of “Department of Biosciences, University Center for Research and Development, Mohali, Punjab, India,” it should be “Department of Biosciences, University Center for Research and Development, Chandigarh University, Mohali, Punjab, India.”

The authors apologize for this error and state that this does not change the scientific conclusions of the article in any way. The original article has been updated.

